# The Application of Transbronchial Lung Cryobiopsy and Uniportal and Tubeless Video-Assisted Thoracic Surgery in the Multidisciplinary Diagnosis of Interstitial Lung disease—A Real-World Prospective Study

**DOI:** 10.3389/fmolb.2021.681669

**Published:** 2021-06-16

**Authors:** Qian Han, Xiaobo Chen, Xin Xu, Weiping Qian, Gui Zhao, Mengmeng Mao, Bingpeng Guo, Shu Xia, Guilin Peng, Jianxing He, Yingying Gu, Shiyue Li, Qun Luo

**Affiliations:** ^1^ Department of Respiratory Medicine, The First Affiliated Hospital of Guangzhou Medical University, Guangzhou, China; ^2^ National Clinical Research Center for Respiratory Disease, Guangzhou Institute of Respiratory Health, Guangzhou, China; ^3^ Department of Cardio-thoracic Surgery, The First Affiliated Hospital of Guangzhou Medical University, Guangzhou, China; ^4^ Department of Pathology, Guangzhou Institute of Respiratory Health, Guangzhou, China

**Keywords:** multidisciplinary diagnosis, interstitial lung disease, cryobiopsy, uniportal and tubeless video-assisted thoracic surgery, pathological diagnosis

## Abstract

The application of transbronchial lung cryobiopsy (TBLC) and uniportal and tubeless video-assisted thoracic surgery (UT-VATS) in the multidisciplinary diagnosis of interstitial lung disease (ILD) has not been demonstrated in real-world clinical practice. This prospective study included 137 patients with no definitive diagnosis who were the subject of two multidisciplinary discussion (MDD) sessions. As indicated in the first MDD, 67 patients underwent UT-VATS and 70 underwent TBLC. The specificity of biopsy information and its contribution to final MDD diagnosis were evaluated in the second MDD. The post-operative complications and hospitalization costs associated with the two biopsy methods were compared. UT-VATS was favored for patients initially diagnosed with idiopathic pulmonary fibrosis (IPF), bronchiolitis-associated interstitial lung disease (RB-ILD)/desquamative interstitial pneumonia (DIP) and undefined idiopathic interstitial pneumonia (UIIP), while TBLC was preferred for pulmonary lymphangioleiomyomatosis (PLAM) and pulmonary alveolar proteinosis (PAP). The spirometry parameters were better in patients who underwent UT-VATS than those who underwent TBLC. UT-VATS provided more specific pathological results than TBLC (85.7 vs 73.7%, *p* = 0.06). In patients initially diagnosed with UIIP, pathological information from UT-VATS was more clinically useful than that obtained from TBLC, although both tests contributed similarly to cases initially diagnosed as interstitial pneumonia with auto-immune features (IPAF)/connective tissue disease-related ILD (CTD-ILD). The safety of UT-VATS was comparable with TBLC although TBLC was cheaper during hospitalization (US$4,855.7 vs US$3,590.9, *p* < 0.001). multidisciplinary discussion decisions about biopsies were driven by current knowledge of sampling and diagnosis capacity as well as potential risks of different biopsy methods. The current MDD considered UT-VATS more informative than TBLC in cases initially diagnosed as UIIP although they were equally valuable in patients initially diagnosed with IPAF/CTD-ILD.

## Introduction

Interstitial lung diseases (ILDs) represent a heterogeneous group of non-neoplastic pulmonary disorders that manifest with varying patterns of parenchymal inflammation and fibrosis of the lungs (Demedts and Costabel, 2002). A specific diagnosis is essential to determine appropriate therapeutic interventions and prognosis. Diagnosis following multidisciplinary discussion (MDD) is considered the gold standard in ILD diagnosis (Flaherty et al., 2004; Chaudhuri et al., 2016; De Sadeleer et al., 2018). Nonetheless MDD pathways are largely heterogeneous due to the absence of specific guidelines. The American Thoracic Society (Raghu et al.)/European Respiratory Society (ERS) joint statement recommends a two-round MDD process and emphasizes the necessity for lung biopsy and utilization of histopathologic information in reaching a final diagnosis (Raghu et al., 2018).

Current guidelines emphasize the significant role of surgical lung biopsy (SLB) in the diagnosis of undefined ILDs, with video-assisted thoracic surgery (VATS) preferred to open thoracotomy (Raghu et al., 2011; Raghu et al., 2018; Travis et al., 2013). Nonetheless traditional VATS is burdened by relatively high morbidity and mortality (Han et al., 2015; Luo et al., 2013; Fibla et al., 2012a; Fibla et al., 2012b; Sigurdsson et al., 2009). The development of uniportal and tubeless VATS (UT-VATS) under spontaneous ventilation anesthesia offers a safe and feasible option in the diagnosis of ILDs (Peng et al., 2017). Recent studies suggest that transbronchial lung cryobiopsy (TBLC) may be an alternative method for obtaining large and well-preserved samples of lung parenchyma. It is less invasive and associated with fewer complications and a lower mortality rate than SLB (Colella et al., 2018; Tomassetti et al., 2016; Johannson et al., 2016).

Little is known about how this two-round MDD model is implemented in real-world practice. It is unclear what proportion of cases require biopsy; and how much pathological information, with different biopsies performed, contributes to the final MDD diagnosis and patient management. In this prospective study, we aimed to evaluate the application of UT-VATS and TBLC, under the current MDD schema, in the diagnostic algorithm of undefined ILDs. The option by the MDD on biopsy methods was described, and the contribution of different biopsy methods in a spectrum of certain ILD subtypes was evaluated by MDD.

## Methods

### Patient Selection and Data Archiving

This was a single center, non-randomized, prospective study that involved consecutive patients referred to the ILD Center, First Affiliated Hospital of Guangzhou Medical University, between June 1st and Dec 1st, 2019. According to the patient triage algorithm at our Center, all referred patients were reviewed at MDD sessions (see below) and the initial diagnosis of ILD was based on clinical-radiological information. Prior to this, complete data (clinical and imaging findings) of each patient were collated.

To ensure integrity and consistency of clinical data, case report forms (CRFs) were used to record patient information, including general information (age, sex, smoking status), exposure history (environmental, occupational, medications), history of physician-diagnosed connective tissue disease (CTD), CTD-related symptoms, signs and serological results, and spirometry. The CRFs were a modified form of the American College of Chest Physicians (ACCP) ILD questionnaire (with CHEST copyright permission) and an ILD questionnaire adopted by the Firestone Institute for Respiratory Health in Canada (by courtesy of MK).

### Study Protocol

The MDD session was held online via a regional research network, and hosted by an expert panel that comprised one pulmonologist (LQ), one respiratory radiologist (ZQS), one thoracic pathologist (GYY) and one rheumatologist (YSH) from our institute, each with recognized expertise in the management of ILDs for at least 10 years.

**FIGURE 1:**
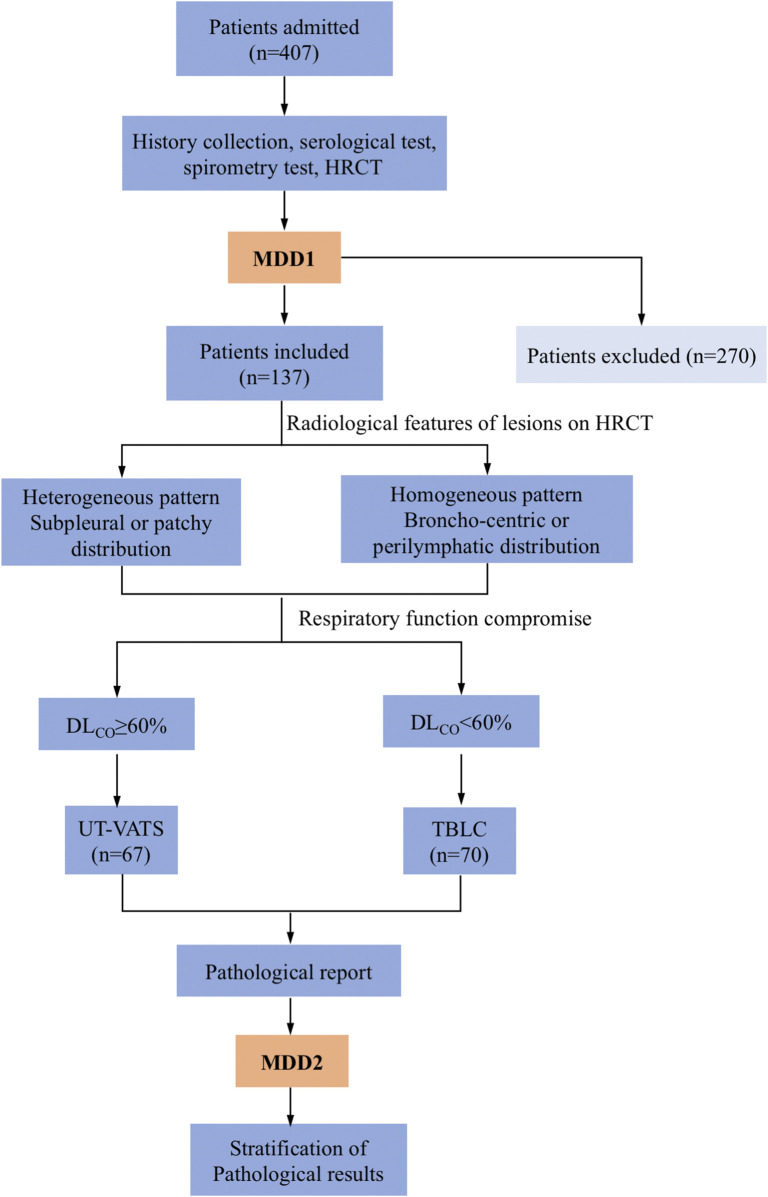
Flowchart of the study. Patients admitted to the institute underwent MDD1 after history collection, serological testing, spirometry and HRCT. A decision was made whether to perform a biopsy and which method (UT-VATS or TBLC) to utilize, the latter based on radiological features of lesions as well as respiratory compromise of patients. MDD2 was held with pathological reports available. Stratification of pathological results was performed based on specificity as well as their contribution to the final MDD diagnosis. MDD: multidisciplinary discussion, HRCT: high resolution computed tomography. UT-VATS: uniportal and tubeless-video-assisted thoracic surgery, TBLC: transbronchial lung cryobiopsy, FVC: forced vital capacity, DL_CO_: carbon-monoxide diffusion coefficient.

As per our practice, two MDD sessions were usually needed to agree a final diagnosis of ILD. The first MDD session produced an initial diagnosis but where histological data were required for accurate diagnosis, a multidisciplinary decision would be made to perform a biopsy with careful scrutiny of the indications for VATS or TBLC based on dominant distribution of lesions on high-resolution CT (HRCT) and spirometry data (Figure 1). All patients gave informed consent. The eligibility and exclusion criteria are described in the supplemental materials (Supplementary Table S1).

Following biopsy, a second MDD session was conducted to review complete clinical, radiological and pathological data and agree a final diagnosis. The evaluation of the pathological diagnosis by the MDD produced one of four grades: Grade 1, histological information was specific to indicate a definitive ILD subtype and sufficient to alter the initial clinical-radiological diagnosis; grade 2, histological information was specific and sufficient to support the initial clinical-radiological diagnosis; grade 3, albeit non-specific, the histological information provided supplementary information for ILD subtype and thereby aided treatment decision, i.e., germinal center formation, lymphoid aggregates, vasculitis; grade 4, the histological information failed to provide diagnostic or therapeutic clues for a given case. The detailed “Pathological evaluation form” is shown in the supplemental materials (Supplementary Table S2).

### Uniportal and Tubeless Video-Assisted Thoracic Surgery and Transbronchial Lung Cryobiopsy

The biopsy location for both procedures was confirmed by the pulmonologist and radiologist on the MDD panel following review of a pre-operative HRCT. Two specimens from different lobes were routinely harvested for UT-VATS; in TBLC, several samples from different segments of the selected lobe were collected. Sample characteristics were evaluated by individual sample size as well as sample number.

The UT-VATS was performed as previously described (Peng et al., 2017) by an experienced cardiothoracic team in our institute (XX and PGL). Briefly, under intravenous anesthesia with a laryngeal mask airway, a 30°/5 mm thoracoscope (Stryker, United States) was inserted into the pleural cavity to exclude any dense or extensive lung-pleura adhesions. Ring-forceps were used to hold the targeted pulmonary segment and the parenchyma gently pulled out of the chest wall. Wedge resection was performed for biopsy and the residual lung tissue sutured. A chest tube was placed into the chest cavity followed by subcutaneous tissue closure. After expansion of the lung through the chest tube and laryngeal mask, the “tubeless” state was realized by rapid removal of the chest tube and subsequent suture of the skin incision.

TBLC was performed by two experienced bronchoscopists (LSY and CXB) under general sedation using an endotracheal tube airway. Samples were obtained using a flexible cryoprobe with a diameter of 1.9 mm (Erbokryo CA, Erbe, Germany) under fluoroscopic guidance. The gas source of the cryosurgical system was CO_2_ with a working pressure of 55–60 bar. The freezing time was 3–6 s, adjusted according to the sample size. Samples were harvested from different bronchopulmonary segments of selected lobes.

The study was registered at clinicaltrials.gov (NCT03958162) and approved by the local independent ethics committee (The First Affiliated Hospital of Guangzhou Medical University; reference number: 2018-85).

### Statistical Analysis

Statistical analyses were performed using IBM SPSS Statistics 20.0 (IBM Corporation, Armonk, NY, United States). Qualitative values are presented as numbers with percentages and compared by Chi-Square test or Fisher’s exact test. Quantitative values are expressed as mean with standard error of mean (SEM) or median with interquartile range and compared by independent t-test or Mann-Whitney test. Kappa coefficient (κ) was used to determine the agreement between pathological results or initial clinical-radiological diagnoses and final diagnoses. A *p* value < 0.05 was considered statistically significant.

## Results

### General Conditions of the Study Population

Between June and Dec 2019, 407 consecutive patients who visited our institute for the first time were initially determined to have ILD. Of these, 137 (33.7%) were eligible and included in this study. As indicated by the first MDD session, 67 patients underwent UT-VATS and 70 underwent TBLC. The two cohorts did not differ significantly in age but there were more female and never-smoking patients in the TBLC compared with the UT-VATS cohort, and spirometry parameters (FVC, FEV1 and DL_CO_%) in the UT-VATS were better than those in the TBLC cohort (Table 1).

**TABLE 1:** Characteristics of patients undergoing UT-VATS or TBLC.

	UT-VATS (*n* = 67)	TBLC (*n* = 70)	*p* value
Age^b^	54.0 (20–70)	50.0 (21–72)	0.27
Male/female, n	39/28	28/42	0.04
Smoking history, no/yes, n	28/39	16/54	0.03
FVC%^a^	81.3 (2.7)	71.8 (2.8)	0.01
FEV1%^a^	80.7 (2.6)	73.0 (2.8)	0.05
DL_CO_%^a^	68.7 (2.4)	47.2 (2.5)	<0.001
Biopsy location, n	–	–	–
LUL	10	NA	–
LL(lingular)L	42	NA	–
LL(lower)L	53	44	–
RUL	1	4	–
RML	9	NA	–
RLL	8	295	–
Sample number, n	–	–	–
1	14 (20.9)	1 (1.4)	–
2	50 (74.6)	1 (1.4)	–
3	3 (4.5)	8 (11.4)	–
4	–	9 (12.9)	–
5	–	31 (44.3)	–
>5	–	20 (28.6)	–
Sample number^b^	2 (1–3)	5 (1–8)	–
Sample size^b^, cm^2^/mm^2^	5.3 (1–32.0)^c^	9.0 (1–40.0)^d^	–

Values are presented as

a mean (SEM)

b median (interquartile range) where appropriate.

c : cm^2^

d : mm^2^

UT-VATS: uniportal and tubeless-video-assisted thoracic surgery, TBLC: transbronchial lung cryobiopsy, FVC: forced vital capacity, FEV1: forced expiratory volume in 1 s, DL_CO_: carbon-monoxide diffusion coefficient. LUL: left upper lobe, RUL: right upper lobe, RML: right middle lobe, RLL: right lower lobe.

### Biopsy Specimens

As shown in Table 1, a total of 123 samples (median: 2, range:1–3) were obtained by UT-VATS and 343 (median: 5, range:1–8) by TBLC. The mean individual sample size was 5.3 (range: 1–32) cm^2^ for UT-VATS and 9.0 (range: 1–40.0) mm^2^ for TBLC. The VATS samples were mainly from the left lobe, especially the left lingular and lower lobes; TBLC samples were mainly from the lower lobes, especially the right lower lobe.

### Multidisciplinary Discussion Choice of Biopsy Methods

The choice of biopsy method and altered MDD diagnosis following the addition of pathological information are listed in Table 2. The multidisciplinary team was inclined to select UT-VATS as the biopsy method where the initial diagnosis was idiopathic pulmonary fibrosis (IPF) (76.9%, 10/13), respiratory bronchiolitis-associated interstitial lung disease (RB-ILD)/desquamative interstitial pneumonia (DIP) (71.4%, 5/7) or unclassifiable idiopathic interstitial pneumonia (UIIP) (59.5%, 22/37); TBLC was more likely to be selected when the initial diagnosis was pulmonary lymphangioleiomyomatosis (PLAM) (100%, 4/4), pulmonary alveolar proteinosis (PAP) (80%, 4/5), connective tissue disease-related ILD (CTD-ILD) (59.1%, 13/22) or interstitial pneumonia with auto-immune features (IPAF) (58.8%, 20/34).

**TABLE 2:** The alteration of MDD diagnosis after the addition of pathological information.

Pre-biopsy dx	Post-biopsy dx		*p* value
	UT-VATS (*n* = 67)	TBLC (*n* = 70)	
IPF (*n* = 13)	IPF 8	IPF 3	0.40
	RBILD/DIP 2	–	
COP (*n* = 1)	NA	COP 1	–
RBILD/DIP (*n* = 7)	RBILD/DIP 2	RBILD/DIP 2	0.15
	IPF 2	–	
	HP 1	–	
LIP (*n* = 2)	NSIP 1	LIP 1	–
UIIP (*n* = 37)	UIIP 9	UIIP 8	0.46
	HP 4	IPF 3	
	IPF 3	NSIP 1	
	RBILD/DIP 2	Pneumoconiosis 1	
	IPAF 1	HP 1	
	LIP 1	ACIF 1	
	Vasculitis 1	–	
	ACIF 1	–	
IPAF (*n* = 34)	IPAF 12	IPAF 20	0.10
	Sarcoidosis 1	–	
–	HP 1	–	
CTD-ILD (*n* = 22)	CTD-ILD 9	CTD-ILD 12	0.43
	–	IPF 1	
HP (*n* = 3)	IPAF 1	HP 1	–
	–	Infection 1	
Sarcoidosis (*n* = 1)	Sarcoidosis 1	NA	–
PAP (*n* = 5)	PAP 1	PAP 4	–
PLAM (*n* = 4)	NA	PLAM 4	–
Vasculitis (*n* = 1)	Vasculitis 1	NA	–
Pneumoconiosis (*n* = 1)	Lipid pneumonia 1	–	–
PLCH (*n* = 2)	UIIP 1	PLCH 1	–
IPH (*n* = 1)	NA	IPH 1	–
PAM (*n* = 1)	NA	PAM 1	–
Infection (*n* = 1)	NA	Infection 1	–

UT-VATS: uniportal and tubeless-video-assisted thoracic surgery, TBLC: transbronchial lung cryobiopsy, IPF: idiopathic pulmonary fibrosis, NSIP: non-specific interstitial pneumonia, COP: cryptogenic organizing pneumonia, RB-ILD: respiratory bronchiolitis interstitial lung disease, DIP: desquamative interstitial pneumonia, LIP: lymphocyte interstitial pneumonia, ACIF: airway-lefted interstitial fibrosis, UIIP: undefined interstitial pneumonia, IPAF: interstitial pneumonia with autoimmune features, CTD-ILD: connective tissue disease-related ILD, HP: hypersensitivity pneumonitis, PAP: pulmonary alveolar proteinosis, PLAM: pulmonary lymphangioleiomyomatosis, PLCH: pulmonary Langerhans cell histiocytosis, IPH: idiopathic pulmonary hemosiderosis, PAM: pulmonary alveolar microlithiasis.

### Pathological Diagnoses

**FIGURE 2:**
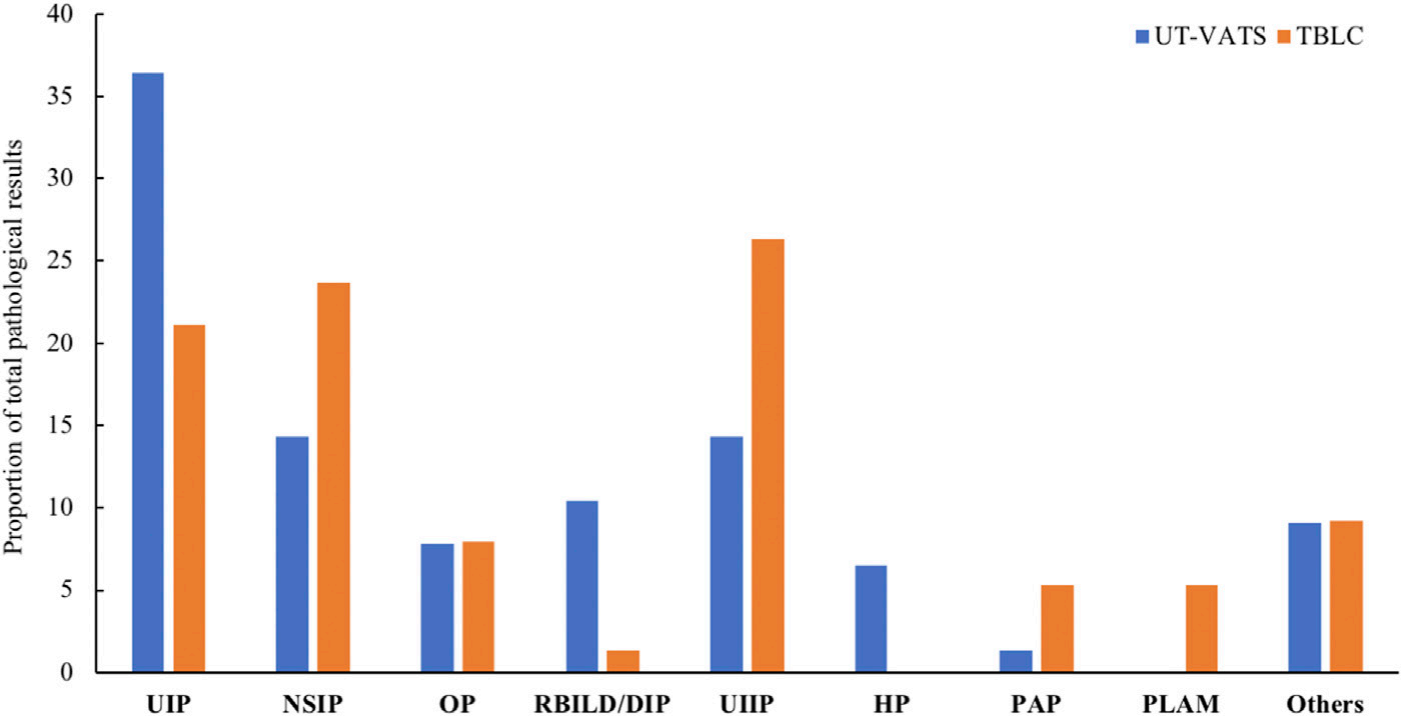
The distribution of pathological diagnoses by different biopsy methods. UT-VATS: uniportal and tubeless-video-assisted thoracic surgery, TBLC: transbronchial lung cryobiopsy, UIP: usual interstitial pneumonia, NSIP: non-specific interstitial pneumonia, OP: organizing pneumonia, RB-ILD: respiratory bronchiolitis interstitial lung disease, DIP: desquamative interstitial pneumonia, UIIP: undefined interstitial pneumonia, HP: hypersensitivity pneumonitis, PAP: pulmonary alveolar proteinosis, PLAM: pulmonary lymphangioleiomyomatosis, Others: including LIP (lymphocyte interstitial pneumonia), sarcoidosis, PLCH (pulmonary Langerhans cell histiocytosis), IPH (idiopathic pulmonary hemosiderosis) and PAM (pulmonary alveolar microlithiasis).

There was a borderline significant increase in specific pathological diagnosis for the UT-VATS compared with TBLC cohort (85.7 vs 73.7%, *p* = 0.06). For UT-VATS, the most common pathological diagnosis was usual interstitial pneumonia (UIP), followed by non-specific interstitial pneumonia (NSIP) and RB-ILD/DIP; for TBLC, UIIP was most commonly encountered, followed by NSIP and UIP (Figure 2). The kappa coefficient between pathological result and final diagnosis was 0.8 (95% CI: 0.7–0.9) for UT-VATS and 0.7 (95% CI: 0.6–0.8) for TBLC.

A total of 42.5% (65 out of 153) of pathological diagnoses were derived from patients diagnosed with IPAF/CTD-ILD, with NSIP as the dominant pattern (38.5%, 25/65), followed by UIP (33.8%, 22/65) (Supplementary Figure S1). The spectrum of connective tissue diseases in this study encompassed idiopathic inflammatory myopathy (IIM), rheumatoid arthritis (RA) and systemic sclerosis (SSc). The distribution of pathological patterns was not significantly different between the two cohorts (*p* = 0.26) (Table 3).

**TABLE 3:** The distribution of pathological diagnosis in IPAF/CTD-ILD patients.

	UT-VATS (*n* = 29)				–	TBLC (*n* = 36)			
–	UIP	NSIP	OP	DIP	UIIP	UIP	NSIP	OP	UIIP
IPAF	8	4	1	1	1	6	11	2	4
IIM-ILD	1	4	2	0	1	1	4	2	1
RA-ILD	3	2	0	0	0	1	0	0	1
SSc-ILD	1	0	0	0	0	1	0	1	1
Total	13	10	3	1	2	9	15	5	7

UT-VATS: uniportal and tubeless-video-assisted thoracic surgery, TBLC: transbronchial lung cryobiopsy. UIP: usual interstitial pneumonia, NSIP: non-specific interstitial pneumonia, OP: organizing pneumonia, DIP: desquamative interstitial pneumonia, UIIP: undefined idiopathic interstitial pneumonia, IPAF: interstitial pneumonia with autoimmune features, IIM-ILD: idiopathic inflammatory myositis-related interstitial lung disease, RA-ILD: rheumatoid arthritis-related interstitial lung disease, SSc-ILD: systemic sclerosis -related interstitial lung disease.

### Stratification of the Pathological Information

As mentioned above, the pathological information was stratified based on its specificity as well as its contribution to the final MDD diagnosis. The grade distribution of pathological results was significantly different for UT-VATS and TBLC (*p* = 0.02): compared with TBLC, UT-VATS was more likely to alter the initial clinical-radiological diagnosis (grade1: 34.3 vs 12.9%, *p* = 0.003) and provide more informative results (grade 4: 9.0 vs 20.0%, *p* = 0.06) to the second MDD session. As shown in Figure 2, among cases with specific pathological diagnoses, those initially diagnosed as UIIP accounted for a majority of diagnosis-altered cases (62.5%, 20/32), followed by RB-ILD/DIP (9.4%, 3/32). Those initially diagnosed as IPAF accounted for a great proportion of diagnosis-unchanged cases (35.1%, 26/74), followed by CTD-ILD (24.3%, 18/74) and IPF (13.5%, 10/74). A subgroup analysis based on ILD subtype and focused on cases initially diagnosed as UIIP and IPAF/CTD-ILD showed that histological information from UT-VATS appeared more informative than that obtained from TBLC for cases initially diagnosed as UIIP (grade 4: 13.6 vs 40.0%, *p* = 0.06). Nonetheless UT-VATS and TBLC contributed similarly to the final diagnosis for cases initially diagnosed as IPAF/CTD-ILD (total *p* = 0.14, grade 4: 8.7 vs 15.2%, *p* = 0.47) (Table 4).

**TABLE 4:** The stratification of pathological information with different biopsy methods

	UT-VATS	TBLC	*p* value
Total, n	67	70	0.02
Grade 1	23 (34.3)	9 (12.9)	0.003
Grade 2	33 (49.3)	41 (58.6)	0.27
Grade 3	5 (7.5)	6 (8.6)	0.81
Grade 4	6 (9.0)	14 (20.0)	0.06
Initial dx UIIP, n	22	15	0.27
Grade 1	13 (59.1)	7 (46.7)	0.46
Grade 2	1 (4.5)	0 (0)	0.40
Grade 3	5 (22.7)	2 (13.3)	0.47
Grade 4	3 (13.6)	6 (40.0)	0.06
Initial dx IPAF/CTD-ILD, n	23	33	0.14
Grade 1	2 (8.7)	0 (0)	0.08
Grade 2	19 (82.6)	25 (75.8)	0.54
Grade 3	0 (0)	3 (9.1)	0.14
Grade 4	2 (8.7)	5 (15.2)	0.47

Values are presented as number (%) unless specified. UT-VATS: uniportal and tubeless-video-assisted thoracic surgery, TBLC: transbronchial lung cryobiopsy, UIIP: undefined interstitial pneumonia, IPAF: interstitial pneumonia with autoimmune features, CTD-ILD: connective tissue disease-related interstitial lung disease. Grade 1: specific diagnosis with final diagnosis altered; grade 2: specific diagnosis without final diagnosis altered; grade 3: non-specific diagnosis with supplementary information provided; grade 4: non-informative diagnosis.

### Safety and Cost

Pneumothorax occurred in 5.7% (4/70) of patients who underwent TBLC. There were no significant differences in severe bleeding, acute exacerbation or 90-days mortality between UT-VATS and TBLC cohorts. The total and post-operative length of hospital stay were comparable for both methods although the hospitalization cost of UT-VATS was much higher (US$4,855.7 vs US$3,590.9, *p* < 0.001) (Table 5).

**TABLE 5:** Complications and hospitalization costs for different biopsy methods.

	UT-VATS (*n* = 67)	TBLC (*n* = 70)	*p* value
Pneumothorax, n	NA	4 (5.7)	NA
Severe bleeding, n	0 (0)	0 (0)	NA
Acute exacerbation, n	2 (3.0)	3 (4.3)	0.68
90-days mortality, n	0 (0)	0 (0)	NA
Hospitalization days^a^	13.0 (4.0)	13.0 (5.5)	0.57
Post-operative hospitalization days^a^	6.0 (3.0)	5.0 (3.0)	0.2
Total expense^a^, US$	4885.7 (1586.0)	3590.9 (2927.0)	0.001

Values are presented as number (%) or

a median (interquartile range).

UT-VATS: uniportal and tubeless-video-assisted thoracic surgery, TBLC: transbronchial lung cryobiopsy.

## Discussion

This prospective study illustrated the two-round MDD decision-making process in the diagnostic algorithm of ILD. The first MDD reflected the choice of experts on candidates and methods of biopsies, and the second revealed the performance of different biopsy methods in patients with distinct ILD subtypes: pathological information via UT-VATS was more clinically useful than that from TBLC for cases initially diagnosed as UIIP, although both methods contributed similarly to cases initially diagnosed as IPAF/CTD-ILD. The safety of UT-VATS was comparable with that of TBLC although the latter appeared to cost less during the hospitalization.

MDD is considered the gold standard in the diagnosis of ILD given its improvement of diagnostic confidence. Although different pathways that follow a multidisciplinary approach to ILD are largely heterogeneous and need further validation, the two-round MDD method is recommended in the ATS/ERS joint statement (Raghu et al., 2018). We implemented this mode in real-world practice and included a pulmonologist, radiologist, pathologist and rheumatologist in the MDD team. The first round MDD aimed to confirm which patients required a biopsy along with the appropriate biopsy method. SLB is regarded as the most reliable tool to provide diagnostic and prognostic information but is associated with substantial morbidity and mortality. In recent years, TBLC has been proposed as an alternative to SLB in the diagnosis of ILD, with a comparable diagnostic yield and less invasive nature (Tomassetti et al., 2016; Johannson et al., 2016; Ravaglia et al., 2016; Kronborg-White et al., 2017), although results are conflicting (Troy et al., 2020; Romagnoli et al., 2019). In the present study, the counterpart of TBLC was uniportal and tubeless VATS (UT-VATS), characterized by spontaneous ventilation anesthesia and absence of postoperative chest tube drainage, thereby mitigating the peri-procedure risk and postoperative discomfort (Peng et al., 2017). Based on current knowledge of the utility and safety of biopsy procedures, our MDD team was inclined to select UT-VATS in cases initially diagnosed as IPF and RB-ILD/DIP; TBLC appeared to be favored more in cases initially diagnosed as PLAM and PAP. This choice may be related to the radiological features of lesions: SLB is preferred in the presence of a heterogeneous pattern with subpleural or patchy distribution, while TBLC is preferred in the presence of a homogeneous pattern with broncho-centric or perilymphatic distribution (Colby et al., 2017).

The major concern raised for TBLC is that a smaller sample from a single locus may not provide the representative data that are obtained from multiple biopsies from multiple lobes via SLB, especially for UIP pattern (Raparia et al., 2016; Patel et al., 2016). Tomassetti et al. reported the comparable change of initial clinical-radiological diagnosis with the addition of histological information from TBLC or SLB (26 vs 36%), and more cases were finally diagnosed as IPF on TBLC (50 vs 39%), albeit with lower inter-pathologist agreement (Tomassetti et al., 2016); similarly, Troy et al. showed a higher proportion of probable UIP relative to UIP pattern in TBLC, while the converse was observed with SLB samples (Troy et al., 2020), indicating that the heterogeneous pattern of IPF may be more reliable on SLB samples. A smoking history combined with typical radiological changes, i.e. bronchial thickening followed by centrilobular nodules and ground-glass opacity (GGO) in RB-ILD and extensive bilateral GGO with a peripheral and basal predominance in DIP, is usually sufficient to diagnose these ILD subtypes causally associated with smoking (Kumar et al., 2018). Nonetheless it should be noted that there is a high chance of overlap of various smoking-related ILDs, such as RB-ILD/DIP and IPF, in which cases confirmation of the dominant lesion is essential for diagnosis orientation and patient management (Bak and Lee, 2017). We identified an altered diagnosis of RB-ILD/DIP and IPF in four cases with UT-VATS samples, raising the possibility that a representative area of the disease or a coexistent fibrotic process could be presented in a higher proportion of SLB samples.

Another concern in decision-making relates to safety. Since low pulmonary function has been suggested to be associated with a higher rate of complications (Ravaglia et al., 2016), the MDD team adopted DL_CO_<45% predicted to exclude biopsy candidates and DL_CO_<60% to refuse UT-VATS. These cut-off values may partly explain the comparable incidence of procedure-related complications between UT-VATS and TBLC groups. The improved surgical technique with less injury and fast track compared with conventional VATS procedure would increase safety for these selected patients (Peng et al., 2017). Among the adverse events related to TBLC, pneumothorax was the most common (5.7%) but the lower incidence compared with many previous studies (Tomassetti et al., 2016; Ravaglia et al., 2016) seems to correlate with the distribution of dominant lesions in the TBLC group, i.e., broncho-centric or perilymphatic distribution. No severe bleeding occurred in our cohort, partly due to intubation with the rigid bronchoscope and prophylactic placement of a Fogarty balloon.

The second round MDD evaluated the pathological information in terms of its specificity as well as its contribution to the final MDD diagnosis. In line with previous data (Colby et al., 2017), the majority of UT-VATS samples were obtained from two lobes and TBLC samples were from different segments of one lobe (mostly lower lobes). There were one to three biopsies with average size of 5.3cm^2^ and one to eight samples with average size of 9.0 mm^2^ in UT-VATS and TBLC groups, respectively. The specific pathological yield in the UT-VATS cohort was borderline higher than that in the TBLC group (85.7 vs 73.7%, *p* = 0.06). The final diagnosis agreed more with UT-VATS than with TBLC (κ = 0.8 vs κ = 0.7), indicating a considerably higher diagnostic confidence for UT-VATS over TBLC.

Apart from histological specificity, biopsy information, albeit non-specific, is valuable if it provides diagnostic clues or suggests a management strategy: the observation of lymphoid aggregates, germinal centers, lymphoplasmacytic inflammation, vasculitis or pleuritis may indicate the underlying “CTD color” (Fischer and Richeldi, 2014), while multiple foci of peribronchiolar metaplasia may favor HP to a great extent (Churg et al., 2017). We therefore stratified the pathological information into four grades based on non-diagnostic supplementary information provided as well as histological specificity, and grades 1–3 were deemed clinically helpful. We found that UT-VATS samples appeared more informative than TBLC samples across the whole ILD spectrum (91.0 vs 80.0%, *p* = 0.06) as well as in cases initially diagnosed as UIIP (86.4 vs 60.0%, *p* = 0.06). These data indicated that following the first round MDD options, pathological information derived from UT-VATS contributed more to the final MDD diagnosis than information obtained from TBLC.

The proportion of patients who underwent biopsy at our institute was relatively higher than previously reported (33.7 vs 7.5–40.3%) (Singh et al., 2017). This may have been attributable to the great number of patients diagnosed with CTD-ILD or IPAF (23/67, 34.3% in UT-VATS and 33/70, 47.1% in TBLC groups, respectively). The necessity for lung biopsy in ILD patients with autoimmune features remains controversial: although the UIP pattern may offer SSc-ILD patients a comparable prognosis to those with a NSIP pattern (Bouros et al., 2002), it may indicate a worse outcome in patients with RA-ILD, IIM-ILD and IPAF (Antoniou et al., 2009) (Kim et al., 2015; Oldham et al., 2016). Recently, it was revealed in the INBUILD trial that nintedanib could slow the decline in FVC in patients with progressive fibrosing ILD other than IPF, and benefits were observed only when those with a UIP-like pattern were grouped together (Flaherty et al., 2019). These results led pulmonologists and rheumatologists to propose a combination therapy of anti-inflammatory and anti-fibrotic agents in patients with autoimmune diseases characterized by a UIP-pattern. In the current study, we found that most histopathological data for IPAF/CTD-ILD patients, albeit specific, did not alter the diagnosis. Although patients with UIP pattern were prescribed the combination therapy mentioned before and closely monitored, the response to therapy and disease behavior are the subject of another study. Regarding biopsy methods, UT-VATS and TBLC contributed similarly to the final MDD diagnosis in providing clinically useful pathological information, and the distribution of histological pattern was similar between UT-VATS and TBLC groups (*p* = 0.26). This may be partly explained by the distribution of lung injury pattern for this subtype: usually symmetrical, basilar and non-subpleural predominant, thus allowing good sampling with TBLC.

There was no significant difference between UT-VATS and TBLC groups in post-operative hospitalization days, although this may be relatively longer compared with previous data because we usually advise our patients to wait for 3–4 days after the procedure for pathological results and a final MDD decision. The hospitalization expense was higher in the UT-VATS group, mainly attributable to the cost of the procedure, so a patient’s ability to pay may also affect choice of biopsy method.

Our study has several limitations. Although it was recommended that the MDD team should decide candidates for biopsy and the biopsy method, there remains no consensus or guideline on how to make the decision. The non-randomized nature of this study may have led to a selection bias. Direct comparison of the techniques is also difficult since they were performed in different patients. Nonetheless our objective was to evaluate TBLC and UT-VATS, each with well-known advantages and disadvantages for sampling and safety, in the real-world clinical practice within the context of MDD. The organizational scheme of this study may provide additional information for the application of biopsy methods in different ILD subtypes, rather than just emphasizing priority or alteration. Second, this study included a limited number of patients so subgroup analysis was difficult to perform in most ILD subtypes. Future studies that focus on a certain subgroup may better inform the application of different biopsy methods under different circumstances. Finally, this study involved patients and MDD experts from only one center, and the counterpart of TBLC in our study was a highly sophisticated surgical technique that has not been widely implemented in other institutes. Results may differ for other institutes. It should be remembered though that improving conventional procedures is just as important, possibly more so, than developing new ones.

## Conclusion

In conclusion, this prospective study demonstrated the option and evaluation of different biopsy methods in the ILD diagnostic algorithm within the two-round MDD context. MDD decisions about biopsies are driven by the current knowledge of risk-benefit of different biopsy methods as well as the working diagnosis of a given patient. Our study provides a robust rationale for future studies investigating the MDD implementation regarding the selection of biopsy methods in ILD diagnostic schema.

## Data Availability

The original contributions presented in the study are included in the article/Supplementary Material, further inquiries can be directed to the corresponding authors.
