# Supplementary figures and images for: The Application of Transbronchial Lung Cryobiopsy and Uniportal and Tubeless Video-Assisted Thoracic Surgery in the Multidisciplinary Diagnosis of Interstitial Lung disease—A Real-World Prospective Study
Source: Front Mol Biosci. 2021 Jun 16;8:681669. doi: 10.3389/fmolb.2021.681669 (PMC8241905; doi:10.3389/fmolb.2021.681669)

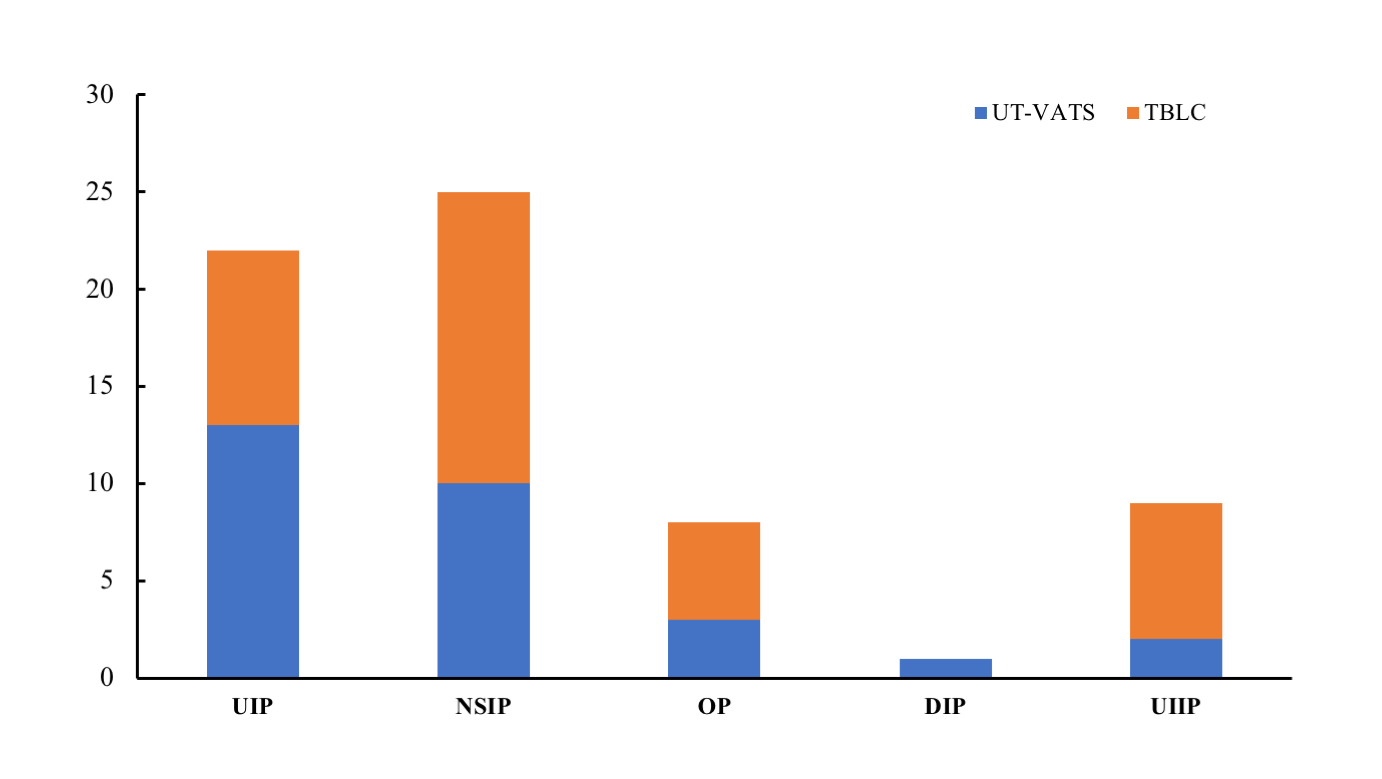

Supplement: Supplementary file 1 [file Image1.TIFF]
