# Supplementary material for: The Application of Transbronchial Lung Cryobiopsy and Uniportal and Tubeless Video-Assisted Thoracic Surgery in the Multidisciplinary Diagnosis of Interstitial Lung disease—A Real-World Prospective Study
Source: Front Mol Biosci. 2021 Jun 16;8:681669. doi: 10.3389/fmolb.2021.681669 (PMC8241905; doi:10.3389/fmolb.2021.681669)
Supplement: Supplementary file 2 [file DataSheet1.DOCX]

**Supplementary Table 1.** Inclusion and exclusion criteria of the study

Inclusion criteria:

1. 18 yrs ≤ age<75yrs

2. Patients diagnosed with interstitial lung disease with comprehensive clinical data available (including history, serological tests, spirometry test and high-resolution computed tomography)

3. Further pathological data still required after multidisciplinary discussion.

Exclusion criteria:

1. Severe pulmonary dysfunction: forced vital capacity (FVC) <65% or carbon-monoxide diffusion coefficient (DLco) <45% of predicted value

2. Pulmonary hypertension: An estimated systolic pulmonary artery pressure >50mmHg on echocardiography

3. Abnormal coagulation parameters or thrombocytopenia (<50 ×10^9^/L)

4. Severe comorbidities leading to an inoperable condition

5. Patient unable to give consent or refuses biopsy

**Supplementary Table 2.** Pathological evaluation form for interstitial lung disease

1. Specific histological pattern

UIP NSIP OP

DAD RB-ILD/DIP LIP

ACIF HP Sarcoidosis

PAP PLAM Vasculitis

Pneumoconiosis PLCH IPH

PAM PPFE AFOP

2. Supplementary histological findings (may indicate different therapeutic means)

2.1 Non-interstitial distribution lesion

bronchiolitis

pleuritis

vasculitis

pulmonary artery hypertension

2.2 Cell infiltration

Germinal center

lymphoid aggregates

numerous lymphocyte infiltration

sparse lymphocyte infiltration

plasma cell infiltration

2.3 Others

emphysema amyloidosis combination

UIP: usual interstitial pneumonia, NSIP: non-specific interstitial pneumonia, OP: organizing pneumonia, RB-ILD: respiratory bronchiolitis interstitial lung disease, DIP: desquamative interstitial pneumonia, LIP: lymphocyte interstitial pneumonia, ACIF: airway-centered interstitial fibrosis, UIIP: undefined interstitial pneumonia, HP: hypersensitivity pneumonitis, PAP: pulmonary alveolar proteinosis, PLAM: pulmonary lymphangioleiomyomatosis, PLCH: pulmonary Langerhans cell histiocytosis, IPH: idiopathic pulmonary hemosiderosis, PAM: pulmonary alveolar microlithiasis, PPFE: pleuroparenchymal fibroelastosis, AFOP: acute fibrinous and organizing pneumonia.

Figure legends

Supplementary figure 1. The distribution of pathological patterns in CTD-ILD/IPAF patients with different biopsy methods.

TBLC: transbronchial lung cryobiopsy, UIP: usual interstitial pneumonia, NSIP: non-specific interstitial pneumonia, OP: organizing pneumonia, DIP: desquamative interstitial pneumonia, UIIP: undefined interstitial pneumonia.
